# Dr. D. D. Thompson, of Ottawa, Ill.

**Published:** 1858-03

**Authors:** 


### DR. D. D. THOMPSON, OF OTTAWA, ILL.

Sometime last year, we published the substance of a communication from the Ottawa Medical Society, by which it appeared that the above-named gentleman was expelled from the society for having embraced homœopathy. In a recent letter to us, he says:

"I am still practising medicine after the old fashion, and shall probably continue to, so long as I practise at all—my *brief* connection with that said *homœopath* to the contrary notwithstanding."

